# Supplementary material for: Identifying the Leading Sources of Saturated Fat and Added Sugar in U.S. Adults
Source: Nutrients. 2024 Jul 30;16(15):2474. doi: 10.3390/nu16152474 (PMC11314151; doi:10.3390/nu16152474)
Supplement: Supplementary file 1 [file nutrients-16-02474-s001.zip › nutrients-3096264-supplementary.pdf]

Supplemental Table S1. Food Categories Contributing to Saturated Fat Intakes

Food	19-30 y	31-50 y	51-70 y	>70 yr	Male	Female	Total
Cheese	8.8%	8.5%	8.1%	6.7%	8.1%	8.5%	8.3%
Pizza	7.8%	5.6%	3.7%	2.2%	6.0%	4.3%	5.2%
Ice cream and frozen dairy desserts	3.3%	3.7%	5.1%	7.9%	4.3%	4.6%	4.4%
Eggs and omelets	3.7%	3.7%	3.9%	4.1%	3.8%	3.8%	3.8%
Burritos and tacos	5.0%	4.1%	2.4%	1.2%	4.0%	2.9%	3.5%
Chicken, whole pieces	3.2%	2.9%	2.4%	1.8%	2.9%	2.5%	2.7%
Butter and animal fats	1.4%	2.3%	3.5%	4.6%	2.4%	3.0%	2.7%
Burgers (single code)	3.7%	2.7%	2.1%	1.2%	3.1%	2.0%	2.6%
Cakes and pies	1.8%	2.5%	2.9%	3.7%	2.3%	2.9%	2.6%
Nuts and seeds	1.6%	2.3%	3.3%	2.7%	2.5%	2.5%	2.5%
Candy containing chocolate	1.9%	2.5%	2.8%	2.4%	2.0%	3.0%	2.4%
Cookies and brownies	2.2%	2.1%	2.5%	3.5%	2.3%	2.5%	2.4%
Meat mixed dishes	1.9%	2.1%	2.6%	2.9%	2.4%	2.2%	2.3%
Milk, reduced fat	2.2%	2.0%	2.1%	3.0%	2.3%	2.1%	2.2%
Beef, excludes ground	2.0%	2.3%	2.0%	1.6%	2.4%	1.6%	2.1%
Doughnuts, sweet rolls, pastries	1.8%	2.0%	2.1%	2.3%	2.0%	1.9%	2.0%
Milk, whole	2.2%	1.9%	1.8%	2.0%	2.0%	1.9%	2.0%
Pasta mixed dishes, excludes macaroni and cheese	2.4%	1.9%	1.7%	1.8%	1.8%	2.1%	1.9%
Sausages	1.4%	1.8%	1.8%	1.8%	2.0%	1.4%	1.7%
Cold cuts and cured meats	1.4%	1.6%	1.9%	2.0%	1.9%	1.4%	1.7%
Other Mexican mixed dishes	2.2%	1.8%	1.1%	0.5%	1.5%	1.6%	1.6%
Cream and cream substitutes	0.8%	1.7%	1.9%	1.7%	1.3%	1.9%	1.6%
Salad dressings and vegetable oils	1.4%	1.5%	1.6%	1.6%	1.3%	1.8%	1.5%
Ground beef	1.7%	1.5%	1.5%	1.1%	1.7%	1.2%	1.5%
French fries and other fried white potatoes	1.9%	1.6%	1.2%	0.8%	1.6%	1.3%	1.5%
Egg/breakfast sandwiches (single code)	1.3%	1.7%	1.2%	0.9%	1.6%	1.1%	1.4%
Soups	1.1%	1.3%	1.4%	1.7%	1.2%	1.5%	1.3%
Macaroni and cheese	1.7%	1.3%	1.1%	1.0%	1.1%	1.5%	1.3%
Poultry mixed dishes	1.1%	1.2%	1.3%	1.3%	1.1%	1.3%	1.2%
Cream cheese, sour cream, whipped cream	1.3%	1.2%	1.2%	1.3%	1.0%	1.4%	1.2%
Pork	1.0%	1.2%	1.2%	1.1%	1.3%	0.9%	1.1%
Yeast breads	1.0%	1.1%	1.2%	1.4%	1.1%	1.2%	1.1%
Dips, gravies, other sauces	0.9%	1.1%	1.0%	1.0%	0.8%	1.2%	1.0%

Biscuits, muffins, quick breads	0.8%	0.8%	1.1%	1.6%	0.9%	1.1%	1.0%
Mashed potatoes and white potato mixtures	0.8%	0.8%	1.1%	1.5%	0.9%	1.0%	1.0%
Potato chips	0.8%	0.9%	0.8%	0.6%	0.8%	0.8%	0.8%
Frankfurters	0.7%	0.8%	0.8%	0.8%	0.9%	0.6%	0.8%
Popcorn	0.7%	0.8%	0.9%	0.6%	0.7%	0.8%	0.8%
Fish	0.6%	0.6%	0.9%	1.0%	0.7%	0.8%	0.7%
Bacon	0.6%	0.7%	0.8%	0.9%	0.7%	0.7%	0.7%
Mayonnaise	0.6%	0.7%	0.7%	0.8%	0.7%	0.6%	0.7%
White potatoes, baked or boiled	*	0.6%	0.8%	1.1%	0.6%	0.7%	0.7%
Frankfurter sandwiches (single code)	0.7%	0.7%	0.7%	0.6%	0.7%	0.6%	0.7%
Tortilla, corn, other chips	0.8%	0.7%	0.5%	*	0.7%	0.6%	0.7%
Other sandwiches (single code)	0.9%	0.6%	0.5%	0.4%	0.7%	0.5%	0.6%
Chicken/turkey sandwiches (single code)	1.1%	0.7%	*	*	0.6%	0.6%	0.6%
Chicken patties, nuggets and tenders	1.0%	0.6%	*	*	0.6%	0.6%	0.6%
Turnovers and other grain-based items	0.7%	0.5%	0.6%	0.5%	0.6%	0.6%	0.6%
Crackers, excludes saltines	0.5%	0.5%	0.6%	0.6%	0.5%	0.7%	0.6%
Margarine	*	*	0.7%	1.3%	0.5%	0.6%	0.5%
Stir-fry and soy-based sauce mixtures	0.6%	0.6%	0.4%	*	0.5%	0.5%	0.5%
Coffee	0.6%	0.6%	*	*	*	0.7%	0.5%
Milk shakes and other dairy drinks	0.5%	0.6%	0.5%	*	0.6%	0.4%	0.5%
Milk, lowfat	*	*	0.4%	0.7%	*	0.5%	0.4%
Rolls and buns	0.5%	*	*	*	*	*	*
Beans, peas, legumes	*	0.4%	0.4%	*	*	*	*
Seafood mixed dishes	*	*	0.5%	0.5%	*	0.4%	*
Tortillas	*	0.5%	*	*	*	*	*
Pancakes, waffles, French toast	*	*	*	0.4%	*	*	*
Other vegetables and combinations	*	*	0.4%	0.4%	*	0.5%	*
Yogurt, regular	*	*	*	*	*	0.5%	*
Oatmeal	*	*	*	0.7%	*	*	*
Vegetable dishes	*	*	*	*	*	*	*
Rice mixed dishes	*	*	*	*	*	*	*
Cheese sandwiches (single code)	*	*	*	0.5%	*	*	*
Fried rice and lo/chow mein	*	*	*	*	*	*	*
Turkey, duck, other poultry	*	*	*	*	*	*	*

Rice	*	*	*	*	*	*	*
Egg rolls, dumplings, sushi	*	*	*	*	*	*	*
Milk substitutes	*	*	*	*	*	*	*
Bean, pea, legume dishes	*	*	*	*	*	*	*

Food	Mexican American	Other Hispanic	Non-Hispanic White	Non-Hispanic Black	Non-Hispanic Asian	Other/Multiracial
Cheese	5.3%	6.9%	8.4%	5.1%	3.5%	7.1%
Pizza	4.3%	5.5%	5.2%	5.4%	4.3%	5.0%
Ice cream and frozen dairy desserts	2.5%	4.1%	4.7%	3.7%	3.3%	3.8%
Eggs and omelets	4.9%	5.1%	3.8%	4.1%	4.3%	3.8%
Burritos and tacos	12.4%	4.5%	4.1%	2.6%	2.5%	4.2%
Chicken, whole pieces	2.4%	3.4%	1.8%	6.4%	3.7%	2.7%
Butter and animal fats	1.1%	1.7%	3.0%	1.4%	1.6%	2.1%
Burgers (single code)	3.2%	3.1%	2.8%	4.4%	1.8%	4.2%
Cakes and pies	1.9%	2.4%	2.6%	3.1%	2.4%	2.9%
Nuts and seeds	1.6%	1.7%	2.9%	1.9%	4.4%	2.5%
Candy containing chocolate	1.2%	1.4%	2.5%	2.1%	2.1%	2.0%
Cookies and brownies	2.6%	2.3%	2.8%	2.9%	2.9%	3.0%
Meat mixed dishes	1.7%	1.6%	2.4%	1.7%	2.2%	2.2%
Milk, reduced fat	2.4%	2.0%	1.9%	1.1%	2.1%	2.2%
Beef, excludes ground	2.4%	2.4%	1.7%	1.9%	1.7%	1.9%
Doughnuts, sweet rolls, pastries	2.7%	2.2%	2.3%	2.2%	2.1%	2.5%
Milk, whole	2.0%	1.9%	1.8%	1.9%	2.6%	1.5%
Pasta mixed dishes, excludes macaroni and cheese	1.4%	2.0%	2.1%	2.4%	1.7%	2.6%
Sausages	1.1%	1.4%	1.6%	2.4%	0.9%	1.5%
Cold cuts and cured meats	0.8%	1.5%	1.7%	1.4%	1.1%	1.6%
Other Mexican mixed dishes	7.2%	3.3%	1.3%	0.5%	0.8%	1.8%
Cream and cream substitutes	1.3%	1.2%	1.7%	1.0%	1.2%	1.4%
Salad dressings and vegetable oils	0.9%	1.1%	1.5%	1.6%	1.1%	1.4%
Ground beef	0.8%	1.0%	1.2%	0.9%	0.6%	1.3%
French fries and other fried white potatoes	1.2%	1.1%	1.2%	1.9%	1.1%	1.5%
Egg/breakfast sandwiches (single code)	3.3%	1.9%	1.5%	2.0%	0.8%	1.8%
Soups	2.1%	1.6%	1.0%	0.9%	4.2%	1.5%
Macaroni and cheese	0.6%	0.7%	1.3%	2.4%	*	1.5%
Poultry mixed dishes	1.1%	0.9%	1.2%	1.0%	1.2%	1.0%
Cream cheese, sour cream, whipped cream	1.0%	1.1%	1.2%	0.5%	0.8%	1.3%
Pork	0.9%	1.3%	0.9%	1.4%	2.1%	1.0%
Yeast breads	0.7%	1.0%	1.1%	1.0%	2.7%	1.3%
Dips, gravies, other sauces	0.7%	*	1.0%	0.8%	0.9%	1.0%
Biscuits, muffins, quick breads	0.6%	0.8%	1.0%	1.6%	1.1%	1.2%

Mashed potatoes and white potato mixtures	0.6%	1.1%	1.2%	1.0%	0.4%	1.0%
Potato chips	*	*	0.6%	1.0%	0.5%	0.6%
Frankfurters	*	*	*	0.4%	*	*
Popcorn	0.6%	0.6%	0.9%	1.1%	0.7%	0.7%
Fish	0.6%	0.8%	0.6%	1.5%	1.7%	0.8%
Bacon	*	0.4%	0.7%	1.1%	0.4%	0.7%
Mayonnaise	0.6%	0.7%	0.7%	0.7%	*	0.7%
White potatoes, baked or boiled	*	0.6%	0.8%	0.5%	*	*
Frankfurter sandwiches (single code)	0.7%	1.0%	1.1%	1.5%	0.5%	1.1%
Tortilla, corn, other chips	0.8%	0.6%	0.6%	0.8%	0.5%	1.1%
Other sandwiches (single code)	0.5%	0.6%	0.7%	0.5%	*	0.6%
Chicken/turkey sandwiches (single code)	0.7%	0.7%	0.6%	1.0%	0.6%	*
Chicken patties, nuggets and tenders	*	0.5%	0.6%	0.9%	0.4%	1.0%
Turnovers and other grain-based items	*	1.1%	0.6%	0.5%	1.3%	0.9%
Crackers, excludes saltines	*	*	0.6%	0.5%	0.4%	0.5%
Margarine	*	*	0.5%	*	*	*
Stir-fry and soy-based sauce mixtures	0.5%	0.5%	0.4%	*	2.0%	0.6%
Coffee	0.6%	1.0%	0.5%	*	0.8%	0.7%
Milk shakes and other dairy drinks	0.5%	0.7%	0.6%	0.6%	0.5%	0.7%
Milk, lowfat	*	*	0.4%	*	*	*
Rolls and buns	*	*	*	*	*	*
Beans, peas, legumes	1.0%	1.0%	*	*	0.6%	*
Seafood mixed dishes	*	*	*	0.5%	0.6%	*
Tortillas	1.6%	0.8%	*	*	0.6%	*
Pancakes, waffles, French toast	*	0.6%	*	0.6%	*	*
Other vegetables and combinations	0.5%	0.7%	*	*	1.1%	*
Yogurt, regular	*	*	*	*	0.8%	*
Oatmeal	*	0.5%	*	*	0.7%	*
Vegetable dishes	*	*	*	*	0.5%	*
Rice mixed dishes	*	0.5%	*	*	0.8%	*
Cheese sandwiches (single code)	*	*	0.5%	*	*	*
Fried rice and lo/chow mein	*	*	*	*	0.9%	*
Turkey, duck, other poultry	*	*	*	*	0.4%	*
Rice	*	0.7%	*	*	0.9%	*

Egg rolls, dumplings, sushi	*	*	*	*	0.8%	*
Milk substitutes	*	*	*	*	0.4%	*
Bean, pea, legume dishes	*	0.7%	*	*	0.4%	*

Supplemental Table S2. Food Categories Contributing to Added Sugars Intakes

**Food**	**19-30 y**	**31-50 y**	**51-70 y**	**>70 yr**	**Male**	**Female**	**Total**
Soft drinks	33.6%	28.9%	19.3%	11.3%	28.8%	22.5%	26.0%
Tea	6.4%	7.0%	7.5%	5.5%	6.6%	7.3%	6.9%
Fruit drinks	8.8%	6.6%	5.7%	5.1%	6.8%	6.8%	6.8%
Cakes and pies	3.9%	6.0%	7.5%	10.8%	5.7%	7.0%	6.3%
Sugars and honey	3.3%	5.4%	6.1%	5.2%	4.9%	5.2%	5.0%
Ice cream and frozen dairy desserts	3.3%	3.8%	5.8%	9.3%	4.5%	4.9%	4.7%
Cookies and brownies	3.6%	3.9%	5.2%	7.6%	4.2%	4.8%	4.5%
Candy containing chocolate	2.4%	3.2%	4.1%	3.6%	2.8%	3.9%	3.3%
Sport and energy drinks	4.5%	3.3%	1.4%	0.6%	4.0%	1.3%	2.8%
Ready-to-eat cereal, higher sugar (>21.2g/100g)	3.2%	2.4%	2.4%	3.2%	2.6%	2.8%	2.7%
Jams, syrups, toppings	2.0%	2.4%	3.1%	4.0%	2.7%	2.5%	2.6%
Candy not containing chocolate	2.2%	2.2%	2.8%	1.8%	2.0%	2.7%	2.3%
Doughnuts, sweet rolls, pastries	1.9%	2.2%	2.4%	2.6%	2.3%	2.1%	2.2%
Yeast breads	1.1%	1.3%	1.9%	2.6%	1.5%	1.6%	1.5%
Cream and cream substitutes	0.8%	1.6%	1.9%	1.4%	1.1%	1.9%	1.4%
Biscuits, muffins, quick breads	1.1%	1.1%	1.6%	2.5%	1.2%	1.6%	1.4%
Liquor and cocktails	1.1%	1.0%	1.1%	0.7%	1.0%	1.2%	1.0%
Yogurt, regular	0.8%	0.8%	1.2%	1.3%	0.6%	1.3%	0.9%
Tomato-based condiments	1.0%	0.9%	0.9%	0.6%	1.1%	0.7%	0.9%
Ready-to-eat cereal, lower sugar (=<21.2g/100g)	0.7%	0.7%	1.0%	1.6%	0.8%	0.9%	0.9%
Coffee	1.1%	0.9%	0.6%	*	0.6%	1.1%	0.8%
Salad dressings and vegetable oils	0.5%	0.7%	1.0%	1.0%	0.7%	0.9%	0.8%
Cereal bars	0.7%	0.8%	0.7%	0.5%	0.6%	0.8%	0.7%
Rolls and buns	0.7%	0.7%	0.8%	0.8%	0.8%	0.6%	0.7%
Gelatins, ices, sorbets	0.5%	0.5%	0.6%	0.7%	0.5%	0.6%	0.5%
Milk shakes and other dairy drinks	*	0.6%	0.5%	*	0.6%	0.5%	0.5%
Pudding	*	0.4%	0.6%	1.1%	0.4%	0.6%	0.5%
Burgers (single code)	0.6%	0.5%	0.4%	*	0.5%	*	0.5%
Oatmeal	*	*	0.5%	0.9%	*	0.5%	0.4%
Pizza	0.5%	*	*	*	0.5%	*	0.4%
Pancakes, waffles, French toast	*	*	*	0.5%	*	0.4%	*
Nutrition bars	*	0.4%	*	*	*	0.4%	*

Meat mixed dishes	*	*	0.4%	0.5%	*	*	*
Milk substitutes	*	*	0.4%	*	*	0.4%	*
Nuts and seeds	*	*	0.4%	*	*	*	*
Nutritional beverages	*	*	*	0.8%	*	*	*
Stir-fry and soy-based sauce mixtures	*	*	*	*	*	*	*
Yogurt, Greek	*	*	0.4%	*	*	0.4%	*
Chicken, whole pieces	*	*	*	*	*	*	*
Beans, peas, legumes	*	*	*	0.5%	*	*	*
Smoothies and grain drinks	*	*	*	*	*	*	*
Flavored milk, nonfat	*	*	*	0.5%	*	*	*
Flavored milk, whole	*	*	*	*	*	*	*
Peaches and nectarines	*	*	*	0.7%	*	*	*
Other fruits and fruit salads	*	*	*	0.5%	*	*	*
Soups	*	*	*	*	*	*	*
Egg rolls, dumplings, sushi	*	*	*	*	*	*	*
Mustard and other condiments	*	*	*	*	*	*	*
Other red and orange vegetables	*	*	*	*	*	*	*

Food	Mexican American	Other Hispanic	Non-Hispanic White	Non-Hispanic Black	Non-Hispanic Asian	Other/Multiracial
Soft drinks	33.6%	27.8%	22.6%	25.3%	15.7%	26.8%
Tea	5.4%	6.2%	8.7%	8.2%	6.1%	11.2%
Fruit drinks	7.6%	9.2%	3.8%	12.0%	5.2%	6.3%
Cakes and pies	4.3%	5.3%	5.9%	5.9%	6.0%	5.6%
Sugars and honey	5.4%	7.9%	4.7%	5.5%	9.0%	5.9%
Ice cream and frozen dairy desserts	2.8%	4.1%	5.2%	3.4%	4.3%	3.0%
Cookies and brownies	4.5%	4.0%	5.1%	4.4%	6.3%	4.7%
Candy containing chocolate	1.8%	1.8%	3.6%	2.6%	3.3%	2.2%
Sport and energy drinks	4.3%	2.7%	3.2%	2.8%	2.1%	3.5%
Ready-to-eat cereal, higher sugar (>21.2g/100g)	2.4%	1.8%	2.8%	2.7%	2.1%	2.7%
Jams, syrups, toppings	1.3%	2.1%	2.9%	2.3%	2.2%	1.7%
Candy not containing chocolate	1.6%	1.2%	2.5%	3.1%	2.2%	2.4%
Doughnuts, sweet rolls, pastries	3.4%	2.2%	2.2%	1.7%	2.7%	2.0%
Yeast breads	1.2%	1.4%	1.5%	1.2%	2.0%	1.3%
Cream and cream substitutes	1.5%	1.3%	1.8%	1.1%	1.4%	2.1%
Biscuits, muffins, quick breads	0.6%	1.0%	1.5%	1.2%	1.8%	0.8%
Liquor and cocktails	0.9%	0.9%	1.5%	1.2%	1.1%	0.9%
Yogurt, regular	0.8%	1.1%	0.9%	*	1.0%	*
Tomato-based condiments	0.7%	0.8%	1.0%	1.0%	0.8%	0.8%
Ready-to-eat cereal, lower sugar (=<21.2g/100g)	0.9%	0.6%	1.0%	*	0.8%	0.5%
Coffee	1.0%	1.5%	0.9%	0.5%	2.3%	1.7%
Salad dressings and vegetable oils	*	*	0.9%	0.6%	0.7%	0.5%
Cereal bars	0.5%	0.7%	0.9%	0.5%	0.7%	0.5%
Rolls and buns	*	0.5%	0.7%	0.4%	0.5%	0.5%
Gelatins, ices, sorbets	0.8%	0.6%	0.4%	0.5%	0.5%	*
Milk shakes and other dairy drinks	*	0.6%	0.6%	0.4%	0.6%	0.5%
Pudding	*	0.8%	0.5%	*	0.8%	0.5%
Burgers (single code)	0.6%	*	0.5%	0.7%	*	0.7%
Oatmeal	*	*	0.4%	*	0.6%	*
Pizza	*	*	*	*	*	*
Pancakes, waffles, French toast	*	*	*	*	*	*
Nutrition bars	*	*	0.6%	*	*	*
Meat mixed dishes	*	*	0.5%	*	0.7%	*
Milk substitutes	0.4%	0.6%	*	*	1.5%	*

Nuts and seeds	*	*	0.5%	*	0.7%	*
Nutritional beverages	*	*	*	*	*	*
Stir-fry and soy-based sauce mixtures	0.5%	*	*	*	0.8%	*
Yogurt, Greek	*	*	0.7%	*	*	*
Chicken, whole pieces	*	*	*	0.6%	0.7%	0.6%
Beans, peas, legumes	*	*	*	*	*	*
Smoothies and grain drinks	1.2%	1.4%	*	*	*	*
Flavored milk, nonfat	*	*	*	*	*	*
Flavored milk, whole	*	*	*	*	0.7%	*
Peaches and nectarines	*	*	*	*	*	*
Other fruits and fruit salads	*	*	*	*	*	*
Soups	*	*	*	*	0.8%	*
Egg rolls, dumplings, sushi	*	*	*	*	0.6%	*
Mustard and other condiments	*	*	*	*	0.6%	*
Other red and orange vegetables	*	*	*	0.5%	*	*

Supplemental Table S3. Percent of the sample reporting the foods or beverages from the Food Categories in the SF/AS food list by the day of record.

	Day 1	Day 2	Both Days			
Food Category	Consumed	Consumed	Not Consumed on either day	Consumed on 1 day	Consumed on both days	Consumed on Any Day
Coffee	53.1%	53.5%	41.1%	16.8%	42.1%	58.9%
Yeast breads	39.3%	41.8%	43.5%	36.3%	20.3%	56.5%
Cheese	36.9%	34.6%	47.9%	36.6%	15.5%	52.1%
Soft drinks	30.9%	27.2%	61.7%	22.1%	16.2%	38.3%
Tea	27.1%	26.9%	64.3%	20.6%	15.1%	35.7%
Eggs and omelets	21.2%	23.5%	66.1%	26.2%	7.8%	33.9%
Chicken, whole pieces	20.1%	21.6%	66.1%	28.5%	5.4%	33.9%
Other vegetables and combinations	20.9%	21.8%	66.7%	26.1%	7.2%	33.3%
Tomato-based condiments	22.2%	18.7%	67.0%	27.6%	5.4%	33.0%
Cookies and brownies	21.8%	19.2%	67.7%	25.6%	6.7%	32.3%
Salad dressings and vegetable oils	20.0%	20.6%	68.1%	25.4%	6.6%	31.9%
Cold cuts and cured meats	19.4%	19.8%	68.7%	25.4%	5.9%	31.3%
Sugars and honey	23.4%	22.1%	70.2%	16.8%	13.0%	29.8%
Nuts and seeds	19.8%	19.2%	71.1%	20.7%	8.2%	28.9%
Cream and cream substitutes	22.8%	22.4%	72.5%	12.3%	15.3%	27.5%
Mustard and other condiments	17.4%	16.3%	72.5%	23.1%	4.3%	27.5%
Rolls and buns	16.8%	15.4%	73.5%	22.7%	3.8%	26.5%
French fries and fried potatoes	16.2%	14.4%	74.5%	22.1%	3.3%	25.5%
Mayonnaise	14.8%	15.0%	76.1%	19.8%	4.1%	23.9%
Ice cream and frozen dairy desserts	15.1%	14.0%	76.8%	18.7%	4.4%	23.2%
Milk, reduced fat	15.4%	15.9%	78.6%	13.2%	8.1%	21.4%
Candy containing chocolate	14.7%	11.9%	78.7%	17.2%	4.1%	21.3%
Soups	12.6%	12.7%	79.1%	18.0%	3.0%	20.9%
Crackers, excludes saltines	13.1%	11.8%	79.8%	16.7%	3.5%	20.2%
Cakes and pies	12.3%	11.6%	79.9%	17.7%	2.4%	20.1%
Potato chips	13.1%	11.1%	80.1%	16.8%	3.0%	19.9%
Tortilla, corn, other chips	13.2%	10.2%	80.5%	16.8%	2.7%	19.5%
Butter and animal fats	11.8%	13.0%	80.6%	15.4%	4.0%	19.4%
Fruit drinks	12.8%	11.9%	80.9%	15.2%	3.9%	19.1%

Meat mixed dishes	11.1%	10.7%	81.2%	17.1%	1.7%	18.8%
Jams, syrups, toppings	11.6%	11.3%	81.5%	15.4%	3.0%	18.5%
Rice	10.8%	12.0%	82.1%	14.6%	3.3%	17.9%
Pizza	10.8%	9.6%	82.3%	16.4%	1.3%	17.7%
Ready-to-eat cereal, higher sugar (>21.2g/100g)	11.2%	11.5%	82.6%	13.5%	4.0%	17.4%
Beef, excludes ground	9.9%	10.0%	82.6%	16.1%	1.3%	17.4%
Dips, gravies, other sauces	11.0%	9.0%	83.0%	15.2%	1.8%	17.0%
Candy not containing chocolate	11.2%	8.8%	83.6%	13.7%	2.7%	16.4%
Pasta mixed dishes, excludes macaroni and cheese	9.4%	8.8%	83.9%	15.0%	1.1%	16.1%
Margarine	10.2%	10.5%	84.0%	12.2%	3.8%	16.0%
Doughnuts, sweet rolls, pastries	10.1%	8.9%	84.2%	13.8%	2.0%	15.8%
Ready-to-eat cereal, lower sugar (=<21.2g/100g)	10.3%	10.9%	84.2%	11.5%	4.3%	15.8%
Mashed potatoes and white potato mixtures	9.0%	8.7%	84.5%	14.3%	1.2%	15.5%
Beans, peas, legumes	9.3%	8.7%	84.9%	13.5%	1.7%	15.1%
Biscuits, muffins, quick breads	8.6%	8.6%	85.6%	12.6%	1.8%	14.4%
Bacon	7.9%	8.2%	86.3%	12.3%	1.4%	13.7%
Fish	7.6%	8.3%	86.3%	12.5%	1.2%	13.7%
Milk, whole	9.7%	9.6%	86.7%	8.5%	4.8%	13.3%
Cream cheese, sour cream, whipped cream	7.6%	6.9%	87.8%	10.8%	1.4%	12.2%
Poultry mixed dishes	6.9%	6.6%	87.9%	11.3%	0.8%	12.1%
Pork	6.8%	6.7%	88.1%	11.0%	0.9%	11.9%
Sausages	6.9%	7.0%	88.2%	10.6%	1.2%	11.8%
Tortillas	7.6%	7.3%	88.6%	8.9%	2.6%	11.4%
Burritos and tacos	6.8%	6.3%	88.7%	10.4%	0.9%	11.3%
Ground beef	6.4%	6.2%	89.0%	10.1%	0.9%	11.0%
White potatoes, baked or boiled	5.4%	6.5%	89.5%	9.8%	0.7%	10.5%
Burgers (single code)	6.3%	5.5%	89.7%	9.6%	0.8%	10.3%
Yogurt, regular	5.9%	7.0%	90.2%	7.3%	2.5%	9.8%
Oatmeal	5.9%	7.0%	90.3%	7.1%	2.6%	9.7%
Popcorn	5.4%	4.7%	91.4%	7.7%	0.9%	8.6%
Milk, lowfat	6.0%	6.5%	91.4%	5.3%	3.3%	8.6%
Liquor and cocktails	6.5%	4.3%	91.6%	6.7%	1.8%	8.4%
Pancakes, waffles, French toast	4.6%	4.9%	92.0%	7.3%	0.7%	8.0%
Rice mixed dishes	4.9%	4.0%	92.1%	7.5%	0.4%	7.9%
Other fruits and fruit salads	4.3%	4.9%	92.3%	6.7%	1.0%	7.7%
Cereal bars	4.4%	4.7%	92.8%	5.9%	1.4%	7.2%
Chicken patties, nuggets and tenders	4.3%	3.7%	92.8%	6.8%	0.4%	7.2%
Sport and energy drinks	4.9%	4.0%	93.0%	5.6%	1.4%	7.0%

Macaroni and cheese	3.9%	3.9%	93.0%	6.7%	0.3%	7.0%
Other Mexican mixed dishes	3.9%	3.5%	93.5%	6.1%	0.4%	6.5%
Seafood mixed dishes	3.6%	3.3%	93.7%	6.0%	0.3%	6.3%
Chicken/turkey sandwiches (single code)	3.5%	3.1%	94.1%	5.5%	0.3%	5.9%
Stir-fry and soy-based sauce mixtures	3.7%	2.8%	94.1%	5.5%	0.3%	5.9%
Egg/breakfast sandwiches (single code)	3.2%	3.5%	94.3%	5.1%	0.5%	5.7%
Peaches and nectarines	3.3%	3.4%	94.5%	4.6%	0.8%	5.5%
Other red and orange vegetables	2.9%	3.2%	94.7%	5.0%	0.4%	5.3%
Fried rice and lo/chow mein	3.0%	2.4%	95.2%	4.6%	0.2%	4.8%
Milk substitutes	3.3%	3.6%	95.3%	2.9%	1.8%	4.7%
Turkey, duck, other poultry	2.7%	2.5%	95.4%	4.3%	0.3%	4.6%
Frankfurters	2.3%	2.6%	95.7%	4.0%	0.3%	4.3%
Vegetable dishes	2.5%	1.8%	96.0%	3.8%	0.2%	4.0%
Turnovers and other grain-based items	2.5%	1.8%	96.1%	3.7%	0.2%	3.9%
Gelatins, ices, sorbets	2.2%	2.1%	96.3%	3.3%	0.4%	3.7%
Other sandwiches (single code)	2.0%	2.2%	96.3%	3.6%	0.1%	3.7%
Egg rolls, dumplings, sushi	2.2%	1.8%	96.4%	3.4%	0.2%	3.6%
Frankfurter sandwiches (single code)	2.1%	1.8%	96.4%	3.5%	0.1%	3.6%
Pudding	2.1%	1.9%	96.5%	3.3%	0.3%	3.5%
Nutrition bars	1.9%	2.1%	96.9%	2.4%	0.7%	3.1%
Yogurt, Greek	2.0%	2.0%	97.0%	2.3%	0.7%	3.0%
Smoothies and grain drinks	1.9%	1.8%	97.1%	2.3%	0.5%	2.9%
Bean, pea, legume dishes	1.4%	1.4%	97.6%	2.3%	0.2%	2.4%
Milk shakes and other dairy drinks	1.3%	1.1%	97.8%	2.2%	0.1%	2.2%
Nutritional beverages	1.4%	1.3%	98.0%	1.4%	0.6%	2.0%
Flavored milk, nonfat	0.8%	0.7%	98.7%	1.2%	0.2%	1.3%
Flavored milk, whole	0.7%	0.6%	98.9%	0.9%	0.1%	1.1%
Cheese sandwiches (single code)	0.5%	0.5%	99.1%	0.9%	0.0%	0.9%

Supplemental Table S4. Nutrient contribution of food list vs total day

	Day 1	n=36,355			Day 2	n=31,756			Average 2 days	n=36,355		
	SF/AS food list	Total	% of day	≥90% from SF/AS food list	SF/AS fod list	Total	% of day	≥90% from SF/AS food list	SF/AS food list	Total	% of day	≥90% from SFAS food list
	Mean (SE)			%	Mean (SE)				Mean (SE)			
Energy (kcal)	1888 (8)	2151 (8)	87.3 (0.1)	52.3%	1780 (9)	2030 (10)	86.9 (0.2)	51.1%	1837 (8)	2097 (8)	87.3 (0.1)	48.5%
Protein (gm)	75.8 (0.4)	83 (0.4)	91 (0.1)	71.5%	74.1 (0.4)	81.2 (0.4)	90.5 (0.1)	70.7%	75 (0.3)	82.3 (0.3)	91.0 (0.1)	69.3%
Carbohydrate (gm)	215 (1)	256 (1)	83 (0.2)	42.4%	203 (1)	244 (1)	81.8 (0.2)	39.9%	209 (1)	250 (1)	82.6 (0.2)	35.7%
Added sugars (tsp. eq.)	17.3 (0.2)	17.8 (0.2)	95 (0.1)	88.2%	15.5 (0.2)	15.9 (0.2)	94.7 (0.2)	88.4%	16.5 (0.2)	17 (0.2)	95.8 (0.1)	88.2%
Dietary fiber (gm)	13.1 (0.1)	16.9 (0.1)	78.1 (0.2)	37.3%	12.9 (0.1)	16.9 (0.1)	76 (0.2)	34.3%	13 (0.09)	16.9 (0.12)	77.3 (0.2)	28.3%
Total fat (gm)	80.1 (0.4)	83.2 (0.4)	95.6 (0.1)	87.3%	75.3 (0.5)	78.3 (0.5)	95.3 (0.1)	86.9%	77.9 (0.4)	81 (0.4)	95.7 (0.1)	87.5%
Saturated fat (gm)	26.5 (0.2)	27.2 (0.2)	96.5 (0.1)	90.3%	24.9 (0.2)	25.6 (0.2)	96.1 (0.1)	89.5%	25.8 (0.1)	26.5 (0.1)	96.7 (0.1)	91.4%
Monounsaturated fat (gm)	28.7 (0.2)	29.6 (0.2)	96.3 (0.1)	88.9%	26.9 (0.2)	27.8 (0.2)	96 (0.1)	88.7%	27.9 (0.1)	28.8 (0.1)	96.4 (0.1)	89.5%
Polyunsaturated fat (gm)	17.9 (0.1)	18.9 (0.1)	94.0 (0.1)	83.1%	16.8 (0.1)	17.8 (0.1)	93.6 (0.1)	82.5%	17.4 (0.1)	18.4 (0.1)	94.1 (0.1)	81.6%
Vitamin E (mg)	7.5 (0.07)	8.6 (0.07)	87.0 (0.2)	59.3%	7.2 (0.07)	8.3 (0.08)	85.9 (0.2)	57.0%	7.3 (0.06)	8.5 (0.07)	86.6 (0.1)	54.1%
Vitamin A, RAE (mcg)	487 (5)	639 (7)	81.4 (0.3)	55.6%	494 (5)	658 (7)	80.1 (0.3)	53.4%	489 (4)	647 (6)	80.3 (0.3)	48.2%
Alpha-carotene (mcg)	*175 (5)*	*401 (12)*	*46.1 (0.4)*	*35.5%*	*185 (6)*	*440 (13)*	*42.3 (0.5)*	*32.0%*	*180 (4)*	*422 (12)*	*47.4 (0.4)*	*30.2%*
Beta-carotene (mcg)	*1056 (25)*	*2230 (47)*	*62.4 (0.4)*	*40.4%*	*1050 (24)*	*2329 (45)*	*59.6 (0.5)*	*37.6%*	*1054 (19)*	*2283 (42)*	*58.5 (0.4)*	*29.6%*
Beta-cryptoxanthin (mcg)	*37.4 (1.4)*	*92.1 (2.1)*	*62 (0.4)*	*51.0%*	*35.8 (1.3)*	*92.1 (1.9)*	*58.4 (0.4)*	*46.8%*	*36.8 (1.3)*	*92.5 (1.8)*	*60.3 (0.4)*	*42.0%*
Lycopene (mcg)	*4037 (68)*	*5309 (82)*	*58.1 (0.4)*	*51.4%*	*3858 (74)*	*5286 (101)*	*54.7 (0.5)*	*48.6%*	*3940 (54)*	*5294 (68)*	*67.8 (0.4)*	*55.0%*
Lutein + zeaxanthin (mcg)	*682 (11)*	*1573 (37)*	*65.7 (0.4)*	*37.7%*	*671 (13)*	*1597 (33)*	*63.3 (0.3)*	*35.0%*	*677 (10)*	*1590 (28)*	*61.9 (0.3)*	*27.0%*
Thiamin (Vitamin B1) (mg)	1.4 (0.01)	1.6 (0.01)	86.5 (0.2)	55.6%	1.4 (0.01)	1.6 (0.01)	85.4 (0.2)	53.4%	1.4 (0.01)	1.6 (0.01)	86.2 (0.2)	50.3%
Riboflavin (Vitamin B2) (mg)	1.9 (0.01)	2.2 (0.01)	86.7 (0.2)	54.0%	1.8 (0.01)	2.1 (0.01)	86.2 (0.2)	53.0%	1.9 (0.01)	2.2 (0.01)	86.6 (0.1)	50.7%

Niacin (mg)	23.1 (0.1)	26 (0.1)	88.9 (0.2)	64.5%	22.7 (0.1)	25.6 (0.1)	88.7 (0.2)	63.8%	22.9 (0.1)	25.9 (0.1)	89.0 (0.1)	62.0%
Vitamin B6 (mg)	1.7 (0.01)	2.1 (0.02)	82.1 (0.2)	43.0%	1.7 (0.01)	2.1 (0.01)	81.8 (0.2)	42.4%	1.7 (0.01)	2.1 (0.01)	82.1 (0.2)	36.7%
Folate, DFE (mcg)	447 (3)	535 (3)	82.4 (0.2)	47.1%	448 (4)	537 (5)	81.3 (0.2)	45.6%	446 (3)	535 (3)	82.2 (0.2)	40.6%
Total choline (mg)	286 (2)	335 (2)	85.8 (0.2)	53.6%	281 (2)	326 (2)	85.9 (0.2)	53.1%	283 (1)	331 (1)	86 (0.2)	49.8%
Vitamin B12 (mcg)	4.7 (0.04)	5.2 (0.05)	92.2 (0.2)	80.8%	4.6 (0.05)	5.2 (0.07)	92.2 (0.2)	81.3%	4.7 (0.03)	5.2 (0.05)	92.2 (0.2)	78.2%
Vitamin C (mg)	38 (0.5)	82.7 (1)	58 (0.4)	31.9%	36.3 (0.5)	83 (1)	55.6 (0.4)	29.7%	37.2 (0.4)	83 (0.9)	54.4 (0.4)	21.2%
Vitamin K (mcg)	63.4 (0.6)	114.9 (2.1)	74.6 (0.3)	46.5%	59.7 (0.6)	113 (2)	72.3 (0.3)	43.7%	61.8 (0.5)	114.6 (1.9)	70.5 (0.3)	35.3%
Calcium (mg)	766 (6)	971 (6)	77.7 (0.2)	32.9%	748 (7)	948 (7)	77.3 (0.2)	33.4%	756 (5)	959 (6)	78.1 (0.2)	28.5%
Phosphorus (mg)	1223 (6)	1388 (7)	88.2 (0.1)	59.4%	1190 (7)	1350 (8)	87.9 (0.1)	58.5%	1207 (6)	1370 (6)	88.2 (0.1)	56.4%
Magnesium (mg)	234 (1)	305 (2)	76.8 (0.2)	23.6%	227 (2)	296 (2)	76.8 (0.2)	23.4%	230 (1)	301 (2)	76.9 (0.2)	17.9%
Iron (mg)	13.1 (0.07)	15 (0.07)	87.1 (0.2)	58.1%	13.2 (0.09)	15.1 (0.09)	86.3 (0.2)	56.4%	13.1 (0.06)	15 (0.07)	87 (0.2)	53.6%
Zinc (mg)	10.5 (0.06)	11.7 (0.07)	90.1 (0.1)	68.6%	10.4 (0.08)	11.5 (0.09)	89.6 (0.1)	66.8%	10.4 (0.06)	11.6 (0.06)	90.2 (0.1)	66.8%
Copper (mg)	1 (0.01)	1.3 (0.01)	76 (0.2)	22.0%	1 (0.01)	1.3 (0.01)	75.4 (0.2)	21.3%	1 (0.01)	1.3 (0.01)	75.9 (0.2)	16.9%
Sodium (mg)	3193 (14)	3542 (15)	89.7 (0.1)	65.0%	3053 (16)	3394 (17)	89.2 (0.2)	63.4%	3127 (13)	3475 (14)	89.7 (0.1)	61.3%
Potassium (mg)	2121 (11)	2691 (14)	79.8 (0.2)	34.4%	2057 (12)	2643 (16)	78.7 (0.2)	32.3%	2089 (10)	2669 (13)	79.1 (0.2)	26.8%
Selenium (mcg)	106 (1)	114 (1)	92.6 (0.1)	78.0%	104 (1)	113 (1)	92 (0.1)	77.5%	105 (0)	113 (1)	92.5 (0.1)	75.0%
Caffeine (mg)	163 (3)	175 (3)	81 (0.4)	76.0%	144 (2)	154 (3)	78.7 (0.4)	73.9%	155 (2)	166 (3)	85.8 (0.3)	79.2%
Alcohol (gm)	3 (0.1)	10.7 (0.3)	6.7 (0.2)	5.7%	1.7 (0.1)	7.5 (0.24)	4.8 (0.2)	4.0%	2.5 (0.1)	9.3 (0.2)	8.2 (0.2)	6.3%

Supplemental Table S5. Complete list of What We Eat in America Food Categories

Milk, whole
Milk, reduced fat
Milk, lowfat
Milk, nonfat
Flavored milk, whole
Flavored milk, reduced fat
Flavored milk, lowfat
Flavored milk, nonfat
Milk shakes and other dairy drinks
Milk substitutes
Cheese
Cottage/ricotta cheese
Yogurt, lowfat and nonfat
Yogurt, regular
Yogurt, Greek
Beef, excludes ground
Ground beef
Pork
Lamb, goat, game
Liver and organ meats
Chicken, whole pieces
Chicken patties, nuggets and tenders
Turkey, duck, other poultry
Fish
Shellfish
Eggs and omelets
Cold cuts and cured meats
Bacon
Frankfurters
Sausages
Beans, peas, legumes
Nuts and seeds
Processed soy products
Meat mixed dishes
Poultry mixed dishes
Seafood mixed dishes
Bean, pea, legume dishes
Vegetable dishes
Rice mixed dishes
Pasta mixed dishes, excludes macaroni and cheese
Macaroni and cheese
Turnovers and other grain-based items
Fried rice and lo/chow mein
Stir-fry and soy-based sauce mixtures
Egg rolls, dumplings, sushi
Burritos and tacos
Nachos
Other Mexican mixed dishes
Pizza
Burgers (single code)
Frankfurter sandwiches (single code)
Chicken/turkey sandwiches (single code)
Egg/breakfast sandwiches (single code)
Other sandwiches (single code)
Cheese sandwiches (single code)
Peanut butter and jelly sandwiches (single code)
Seafood sandwiches (single code)
Soups
Rice
Pasta, noodles, cooked grains
Yeast breads
Rolls and buns
Bagels and English muffins
Tortillas
Biscuits, muffins, quick breads
Pancakes, waffles, French toast
Ready-to-eat cereal, higher sugar (>21.2g/100g)
Ready-to-eat cereal, lower sugar (=<21.2g/100g)
Oatmeal
Grits and other cooked cereals
Potato chips
Tortilla, corn, other chips
Popcorn
Pretzels/snack mix
Crackers, excludes saltines
Saltine crackers
Cereal bars
Nutrition bars
Cakes and pies
Cookies and brownies
Doughnuts, sweet rolls, pastries
Candy containing chocolate
Candy not containing chocolate
Ice cream and frozen dairy desserts
Pudding
Gelatins, ices, sorbets
Apples
Bananas
Grapes
Peaches and nectarines

Strawberries
Blueberries and other berries
Citrus fruits
Melons
Dried fruits
Other fruits and fruit salads
Pears
Pineapple
Mango and papaya
Tomatoes
Carrots
Other red and orange vegetables
Broccoli
Spinach
Lettuce and lettuce salads
Other dark green vegetables
String beans
Cabbage
Onions
Corn
Other starchy vegetables
Other vegetables and combinations
Fried vegetables
Coleslaw, non-lettuce salads
Vegetables on a sandwich
White potatoes, baked or boiled
French fries and other fried white potatoes
Mashed potatoes and white potato mixtures
Citrus juice
Apple juice
Other fruit juice
Vegetable juice
Diet soft drinks
Diet sport and energy drinks
Other diet drinks
Soft drinks
Fruit drinks
Sport and energy drinks
Nutritional beverages
Smoothies and grain drinks
Coffee
Tea
Beer
Wine
Liquor and cocktails
Tap water
Bottled water
Flavored or carbonated water
Enhanced or fortified water
Butter and animal fats
Margarine
Cream cheese, sour cream, whipped cream
Cream and cream substitutes
Mayonnaise
Salad dressings and vegetable oils
Tomato-based condiments
Soy-based condiments
Mustard and other condiments
Olives, pickles, pickled vegetables
Pasta sauces, tomato-based
Dips, gravies, other sauces
Sugars and honey
Sugar substitutes
Jams, syrups, toppings
Baby food: cereals
Baby food: fruit
Baby food: vegetable
Baby food: meat and dinners
Baby food: yogurt
Baby food: snacks and sweets
Baby juice
Baby water
Formula, ready-to-feed
Formula, prepared from powder
Formula, prepared from concentrate
Human milk
Protein and nutritional powders
Not included in a food category
